# Herd protection of unvaccinated adults by oral cholera vaccines in rural Bangladesh

**DOI:** 10.1093/inthealth/ihy085

**Published:** 2018-11-29

**Authors:** Mohammad Ali, Peter Kim, K Zaman, John Clemens

**Affiliations:** 1 Johns Hopkins Bloomberg School of Public Health, Baltimore, MD, USA; 2 International Centre for Diarrhoeal Disease Research, Bangladesh, Dhaka, Bangladesh; 3 Chadwick School, Songdo-dong, South Korea; 4 UCLA Fielding School of Public Health, Los Angeles, CA, USA; 5 Korea University College of Medicine, Seoul, South Korea

**Keywords:** cholera, herd protection, oral cholera vaccine

## Abstract

**Background:**

Past research has suggested that the most cost-effective approach to using oral cholera vaccines (OCVs) to control endemic cholera may be to target only children <15 y of age. However, the assumption that vaccination of children with OCVs protects unvaccinated adults has never been tested.

**Methods:**

We reanalyzed the data of an OCV trial in Bangladesh in which children 2–15 y of age and women >15 y of age were allocated to OCV or placebo and assessed herd protection by relating the risk of cholera in each nonvaccinated adult (>15 y) to OCV coverage (OCVC) of residents residing in virtual clusters within 500 m of the residence of that unvaccinated adult.

**Results:**

The risk of cholera in unvaccinated adults decreased by 14% with each 10% increase of OCVC of all targeted age groups (95% 7 to 21%, p=0.0004). Also, the risk of cholera in unvaccinated adults decreased by 13% with each 10% increase in OCVC of children 2–15 y of age (95% CI 6 to 20%, p=0.0007). A high correlation between levels of OCVC of children and adult females precluded an assessment of the herd protection of unvaccinated adults by vaccinating children <16 y of age, independent of concomitant vaccination of adult women.

**Conclusions:**

Unvaccinated adults benefitted from herd protection conferred by OCVs in this trial. Vaccination of children may be sufficient to confer this protection, but this possibility needs to be evaluated in further studies.

## Introduction

Inactivated oral cholera vaccines (OCVs) are now recommended by the World Health Organization (WHO) to help control cholera in both epidemic and endemic settings in conjunction with other cholera prevention and control strategies, such as clean water and sanitation.^1^ To facilitate the use of these vaccines in such settings, since 2013, Gavi, the Vaccine Alliance, has supported a global OCV stockpile, with the WHO as the stockpile secretariat.^2^ The number of doses of OCV deployed from the stockpile has increased almost exponentially with each year since the stockpile’s creation. Between July 2013 and September 2017, close to 17 million OCV doses were shipped to 18 countries in Africa, Asia and the Americas.^3^

To date, the stockpile has been used to a greater extent in cholera epidemics, often in the context of complex humanitarian emergencies, than for control of endemic cholera, despite the fact that endemic cholera accounts for the vast majority of cholera cases and deaths globally.^4^ This disparity in deployment of OCV doses stems in part from the enormity of the population at risk for endemic cholera, estimated at 1.3 billion people, which is well beyond the current or expected global capacity for producing OCV.^4,5^ Another consideration is the cost-effectiveness of using OCVs in such settings: the use of these vaccines is projected to be considerably more cost effective when vaccination is targeted to children <15 y of age rather than to the general population.^6^ However, these cost-effectiveness analyses have assumed that vaccination of children will also protect adults via vaccine herd protection, an assumption that has yet to be verified. In this article we reanalyze the 1985 trial of inactivated OCVs in Bangladesh, in which each of two tested OCVs conferred approximately 60% protection over the first year of surveillance, to assess whether unvaccinated adults were indeed protected via vaccine herd protection.

## Methods

### Overview

We analyzed the vaccine herd protective effects of two inactivated OCVs in a placebo-controlled, individually randomized trial done in the 1980s in Matlab, a rural area of Bangladesh.^7,8^ To analyze these effects, we defined ‘virtual clusters’ as persons whose residences were within a specified radius of the residence of each person (focal person) under analysis. We then related the risk of cholera for each focal person to the OCV coverage of persons in the surrounding virtual cluster. An inverse relationship for focal persons who had not received OCV suggested indirect vaccine herd protection of these unvaccinated persons.^9,10^

### The Matlab OCV trial

The Matlab field studies area of the International Centre for Diarrhoeal Disease Research, Bangladesh (now called the icddr,b) had a total population of approximately 190 000 persons at the time of the trial. As described in detail elsewhere,^7,8^ in the Matlab trial, eligible persons (children aged 2–15 y and nonpregnant women >15 y) were individually randomized to a three-dose regimen of an oral cholera toxin B subunit, killed whole cell (BS-WC) vaccine, oral killed whole cell-only (WC) vaccine or oral placebo. Dosing with inactivated OCV or placebo was conducted between January and May 1985. In total, 89 596 eligible subjects received at least one dose of vaccine or placebo.

Surveillance was conducted for all diarrheal patients from the study area who came either to the icddr,b Matlab hospital or two community-operated treatment centers. A diarrheal visit was defined as the presence of at least three loose or liquid motions in the 24 h before presentation or one to two or an indeterminate number of loose or liquid stools in the 24 h before presentation with at least two signs of dehydration (poor skin turgor, sunken eyes, dry mucous membranes, weakened radial pulse) on presentation. Stools or rectal swabs were collected from these patients and were tested for *Vibrio cholerae* O1 using conventional microbiological methods.^11,12^ Diarrheal visits for which the onset of symptoms was ≤7 d after discharge for the previous diarrheal visit were grouped into single diarrheal episodes. A cholera episode was defined as a diarrheal episode in which no constituent visit was for bloody diarrhea and in which *V. cholerae* O1 was isolated from a fecal specimen from at least one constituent visit.

### OCV coverage (OCVC) of residents in virtual clusters

To assess the herd protection of the OCVs under study, we needed to ascertain the OCVC of residents in virtual clusters constructed around each analyzed individual. With the use of geospatial coordinates ascertained for residences of the entire study population, we constructed a virtual cluster around the residence of each adult, using an earlier described statistical criterion to define a common radius (500 m). The virtual cluster was therefore formed with the individuals living within 500 m for each analyzed individual. As earlier described, this criterion was related to the variability of the variances of vaccine coverage across virtual clusters.^9^ OCVC levels for each analyzed age group (children 2–15 y, females >15 y or children and females combined, depending on the analysis) were calculated as the number of recipients of at least two doses of either OCV divided by the number of eligible residents living within the cluster at the time of the first dose.

### Analytic strategies

In the present analyses, we considered cholera episodes occurring during the period 1 June 1985–31 May 1986, an interval in which OCV herd protection was demonstrable in our earlier analyses,^9^ and following our previous analyses, we defined a person as vaccinated if she/he received at least two doses of either OCV, which, because of the similarity of their constituents and protective efficacy, were combined for this analysis. Indirect vaccine protection was assessed by relating the risk of cholera for each unvaccinated adult during the year of follow-up to the OCVC in the surrounding virtual cluster. In simple analyses, we evaluated whether there was an inverse, monotonic relationship between the risk of cholera in each unvaccinated adult and the OCVC, arranging all unvaccinated adults under analysis and their corresponding levels of OCVC into approximate quintiles of OCV coverage for the entire adult population. We used the Cochrane–Armitage test to statistically assess the trend of the risk of cholera with increasing levels of OCVC. In multivariable logistic regression models, we assessed the relationship between the levels of OCVC, expressed dimensionally and fitted as an independent variable, and the occurrence of cholera, the dependent variable, after controlling for potentially confounding variables. We expressed vaccine coverage as a dimensional variable in these models after first confirming that the relationship between vaccine coverage and the log odds of cholera was roughly linear. Because individuals in Matlab live in geographical clusters of households, termed *baris*, we used generalized estimating equations with exchangeable matrices to adjust for clustering by *bari* in our models. We included as covariates the following variables related to the analyzed adult: age (in years, ascertained at the inception of the trial), sex, religion (Muslim vs other), distance from the residence to the nearest river (in kilometers), distance from the residence to the nearest cholera surveillance site (in kilometers) and occurrence of bloody diarrhea during the 1 y of follow-up. The last variable was included to help reduce residual confounding after controlling for the other covariates, since dysentery, which should not be prevented by OCV, shares several risk factors with cholera.^9^

Multivariable relative risks (estimated by odds ratios relating each percent increase in vaccine coverage to the occurrence of cholera) were estimated by exponentiation of the coefficient for the vaccine coverage variable in the models. The percentage decrease in the risk of cholera associated with each percent increase of OCVC in the surrounding cluster was calculated as (1−multivariable relative risk)×100%. For ease of interpretation, we present the percentage reduction of risk associated with each 10% increase in OCVC. The p-values and 95% CIs for these multivariable relative risks were estimated with the use of the standard errors of these coefficients. All p-values and 95% CIs were two-sided.

## Results

There were 107 465 persons over the age of 15 y at the onset of the trial. Of these, 20 243 were vaccinated females, 34 189 were unvaccinated females and 53 033 were males, all of whom were unvaccinated. In virtual clusters surrounding all unvaccinated persons >15 y of age, vaccine coverage (mean±standard deviation) was 40±12% among targeted women and children (2–15 y of age) combined, 37±11% among targeted women and 43±12% among targeted children. During the first year of follow-up, 472 persons >15 y of age developed cholera (4.4 cases per 1000). Among the 87 222 unvaccinated individuals, 440 cholera cases were detected (5.1 cases per 1000)—213 (6.3 cases per 1000) in unvaccinated females and 227 (4.3 cases per 1000) in unvaccinated males.

We first examined the association between the risk of cholera in unvaccinated persons and approximate quintiles of OCVC of the surrounding age-targeted population (persons 2–15 y of age and females >15 y). As shown in Table 1, the risk among all unvaccinated adults declined from 7.0 per 1000 in the lowest OCVC quintile to 3.6 per 1000 in the highest OCVC quintile (p<0.0001 for trend). This decline was seen in both unvaccinated women and men, but was more pronounced among unvaccinated women. It should be mentioned that the odds of having cholera among adult women in relation to adult men was 1.44 (95% CI 1.19 to 1.74, p=0.0001) in the study area. We also calculated the multivariable relative risk among persons living in progressively higher coverage strata. After controlling for potential confounding variables in the multivariable models, the decline in the risk of cholera for each 10% increase of OCVC was 14% (95% CI 7 to 21, p=0.0004) for all unvaccinated adults, 21% (95% CI 11 to 30, p=0.0001) for unvaccinated women and 8% (95% CI −2 to 17, p=0 0.14) for unvaccinated men. Interaction terms for vaccine coverage and for gender of the unvaccinated adult in the models revealed a significantly greater impact of vaccine coverage on the risk of cholera in unvaccinated women than in unvaccinated men (p<0.05).

**Table 1.** Risk of cholera in unvaccinated adults by OCVC of targeted children and adult females in during a 1-y postvaccination period, Matlab, Bangladesh

OCVC^a^	All unvaccinated adults			Unvaccinated women			Unvaccinated men		
	n^b^	Cases (risk)^c,d^	Relative risk (p-value)^f^	n^b^	Cases (risk) ^c,d^	Relative risk (p-value)^f^	n^b^	Cases (risk)^c,e^	Relative risk (p-value)^f^
<28%	19 529	137 (7.0)	–	8734	81 (9.3)	–	10 868	56 (5.2)	–
28–35%	18 458	107 (5.7)	0.82 (0.13)	7722	50 (6.5)	0.70 (0.04)	10 863	57 (5.2)	1.02 (0.92)
36–40%	17 175	92 (5.4)	0.76 (0.04)	6822	39 (5.7)	0.61 (0.01)	10 511	53 (5.0)	0.98 (0.90)
41–46%	16 671	52 (3.1)	0.44 (<0.0001)	6196	27 (4.3)	0.47 (0.0007)	10 683	25 (2.4)	0.45 (0.001)
>46%	14 596	52 (3.6)	0.51 (<0.0001)	4715	17 (3.6)	0.39 (0.0004)	10 108	35 (3.6)	0.67 (0.06)

^a^OCVC of children aged 2–15 y and females >15 y. The categories reflect approximate quintiles of OCVC of children aged 2–15 y and females >15 y for persons >15 y of age.

^b^Total number of residents in the cited category (all unvaccinated adults, unvaccinated women or unvaccinated men).

^c^Number of cholera cases and risk of cholera per 1000 detected among persons in the cited category during the first year of follow-up after dosing.

^d^p<0.0001 for trend.

^e^p=0.0010 for trend.

^f^Relative risk of cholera among persons living in the cited OCVC quintile compared with persons living in the lowest OCVC area (<28%).

We next assessed the association between the quintile of OCVC of surrounding children 2–15 y of age and the risk of cholera in unvaccinated adults (Table 2). Simple analyses revealed significant inverse relationships for the risk of cholera among all unvaccinated adults (p<0.0001), unvaccinated women (p<0.0001) and unvaccinated men (p=0.0214). As in the analyses of OCVC of women and children combined, in multivariable models the OCVC of children was significantly associated in an inverse fashion with the risk of cholera in all unvaccinated adults and unvaccinated women, but not unvaccinated men, with declines of risk with each 10% increase in OCVC of 13% (95% CI 6 to 20, p=0.0007) in all unvaccinated adults, 20% (95% CI 10 to 29, p=0.0001) in unvaccinated women and 7% (95% CI −3 to 16, p=0.19) in unvaccinated men. Interaction terms for vaccine coverage and for gender of the unvaccinated adult in the models revealed a significantly greater impact of vaccine coverage on the risk of cholera in unvaccinated women than in unvaccinated men (p<0.05).

**Table 2.** Risk of cholera in unvaccinated adults by OCVC of targeted children during the 1-y postvaccination period, Matlab, Bangladesh

OCVC^a^	All unvaccinated adults			Unvaccinated women			Unvaccinated men		
	n^b^	Cases (risk)^c,d^	Relative risk (p-value)^f^	n^b^	Cases (risk)^c,d^	Relative risk (p-value)^f^	n^b^	Cases (risk)^c,e^	Relative risk (p-value)^f^
<32%	18 529	126 (6.8)	–	8275	80 (9.7)	–	10 320	46 (4.4)	–
32–43%	20 246	127 (6.3)	0.92 (0.52)	8421	54 (6.3)	0.66 (0.02)	11 957	73 (6.3)	1.37 (0.09)
44–48%	16 668	63 (3.8)	0.55 (0.0001)	6588	29 (4.4)	0.46 (0.0003)	10 222	34 (3.3)	0.75 (0.19)
49–53%	15 991	70 (4.4)	0.64 (0.003)	6001	31 (5.2)	0.53 (0.003)	10 202	39 (3.8)	0.85 (0.48)
>53%	14 995	54 (3.6)	0.53 (0.0001)	4904	20 (4.1)	0.42 (0.0005)	10 332	34 (3.4)	0.73 (0.18)

^a^OCVC of children aged 2–15 y. The categories reflect approximate quintiles of OCVC of children aged 2–15 y for persons >15 y of age.

^b^Total number of residents in the cited category (all unvaccinated adults, unvaccinated women or unvaccinated men).

^c^Number of cholera cases and risk of cholera per 1000 detected among persons in the cited category during the first year of follow-up after dosing.

^d^p<0.0001 for trend.

^e^p=0.0214 for trend.

^f^Relative risk of cholera among persons living in the cited OCVC quintile compared with persons living in the lowest OCVC area (<32%).

We then analyzed the association between the quintile of OCVC of surrounding women >15 y of age and the risk of cholera in unvaccinated adults (Table 3). In simple analyses there were significant inverse relationships for the risk of cholera among all unvaccinated adults (p<0.0001), unvaccinated women (p<0.0001) and unvaccinated men (p=0.0066). Multivariable models assessing reductions of the risk with every 10% increase of OCVC again found significant reductions in risk in all unvaccinated adults (15% [95% CI 7 to 22], p=0.0004) and unvaccinated women (22% [95% CI 11 to 31], p=0.0002), but not in unvaccinated men (9% [95% CI −2 to 19], p=0.09). Interaction terms for vaccine coverage and for gender of the unvaccinated adult in the models revealed a suggestively greater impact of vaccine coverage on the risk of cholera in women than in men (p=0.06).

**Table 3.** Risk of cholera in unvaccinated adults by OCVC of targeted women only during the 1-y postvaccination period, Matlab, Bangladesh

OCVC^a^	All unvaccinated adults			Unvaccinated women			Unvaccinated men		
	n^b^	Cases (risk)^c,d^	Relative risk (p-value)^f^	n^b^	Cases (risk)^c,d^	Relative risk (p-value)^f^	n^b^	Cases (risk)^c,e^	Relative risk (p-value)^f^
<27%	19 092	128 (6.7)	–	8635	79 (9.1)	–	10 526	49 (4.7)	–
27–34%	18 037	110 (6.1)	0.90 (0.46)	7610	51 (6.7)	0.73 (0.08)	10 543	59 (5.6)	1.02 (0.34)
35–40%	16 798	83 (4.9)	0.74 (0.03)	6646	33 (5.0)	0.54 (0.003)	10 314	50 (5.8)	1.04 (0.84)
41–46%	16 728	67 (4.0)	0.59 (0.0006)	6169	33 (5.3)	0.58 (0.009)	10 767	34(3.3)	0.68 (0.08)
>46%	15 774	52 (3.3)	0.49 (<0.0001)	5129	18 (3.5)	0.38 (0.0002)	10 883	34 (3.1)	0.67 (0.07)

^a^OCVC of females >15 y of age. The categories reflect approximate quintiles of OCVC of females >15 y of age for persons >15 y of age.

^b^Total number of residents in the cited category (all unvaccinated adults, unvaccinated women or unvaccinated men).

^c^Number of cholera cases and risk of cholera per 1000 detected among persons in the cited category during the first year of follow-up after dosing.

^d^p<0.0001 for trend.

^e^p=0.0066 for trend.

^f^Relative risk of cholera among persons living in the cited OCVC quintile compared with persons living in the lowest OCVC area (<27%).

In multivariable models that included independent variables for OCVC of children and of adult women, neither was independently associated with the risk of cholera in all unvaccinated adults, unvaccinated women or unvaccinated men due to the high correlation between levels of OCVC among children and among women within the virtual clusters (correlation coefficient>0.90 for virtual clusters around all unvaccinated adults, unvaccinated women and unvaccinated men).

## Discussion

Our data demonstrate that combined vaccination of children and adult women with inactivated OCVs was associated with indirect vaccine herd protection of unvaccinated adults in Matlab, Bangladesh. Although this herd protection applied to both unvaccinated men and unvaccinated women in simple analyses, significant vaccine herd protection was seen only in adult women after adjustment for potentially confounding variables. It is interesting that we were unable to identify significant OCV herd protection of adult men in the population in our multivariable analyses. This may be due to differences in transmission patterns. Whereas women and children are more likely to acquire cholera from person-to-person transmission in the home, adults males are thought to acquire cholera outside the home, often in the context of occupations such as fishing.^13,14^ If this is true, one would not expect OCVC of the residents in the surrounding environment to protect adult males.

While our study was unable to dissect the herd protective effects of vaccinating children independent of also vaccinating adult women, the analyses did show substantial herd protection of unvaccinated adults associated with vaccination of nearby children and adult women. These findings are consistent with overall analyses of herd protection by OCVs in several sites, including Kolkata^15^ and Zanzibar.^16^ OCVC of children per se was associated with significant protection of adult women in both simple and multivariable analyses but was highly correlated with OCVC of women residing in the same virtual clusters, so the herd effects of vaccine coverage of children independent of OCVC of adult women could not be properly evaluated.

Several limitations of our study warrant discussion. Our estimates of the reduction of risk of cholera with increased OCVC were based on the vaccine coverage and cholera surveillance data from the Matlab trial and may not extrapolate beyond the ranges of coverage observed in the trial. In addition, our study was conducted in a population with endemic cholera, so the results cannot readily be extrapolated to cholera outbreaks in cholera-naïve populations. Also, the study was conducted three decades ago, when the circulating strains of cholera included *V. cholerae* O1 of both the classical and El Tor biotypes. Contemporary strains in rural Bangladesh are El Tor hybrids, in which the El Tor phenotype strains produce classical biotype cholera toxin. Such hybrid strains had not yet emerged at the time of the OCV trial under analysis.^17^ However, because an inactivated OCV very similar to the vaccines tested in Bangladesh conferred both direct and herd protection against hybrid El Tor strains when tested in Kolkata, we do not believe that this constitutes a significant limitation.^15,18^ Moreover, although the design of the vaccine trial was individually randomized, the analyses of associations of vaccine coverage with the risk of cholera were not randomized and the associations could have been distorted by confounding bias. However, we believe that this bias was limited in our study, as we controlled for major known risk factors for cholera in the Matlab population^19^ and we additionally controlled for whether each subject developed dysentery, a syndrome not caused by cholera, yet one that has multiple risk factors in common with cholera.

## Conclusions

Our study lends support to the assertion that vaccination of children with OCVs may confer herd protection to unvaccinated adults in populations with endemic cholera. However, because vaccination of children was confounded with vaccination of adult women in this study, our findings can only be considered suggestive. Such herd protection needs to be evaluated in further studies in which only children ≤15 y of age are vaccinated.

## Authors’ contributions

MA and JC contributed to the study design and implementation. MA, JC and KZ contributed to the implementation and supervision of the study. MA and PK were involved in data accumulation and analysis, and the accuracy of the data analysis. All authors participated in the writing of the manuscript.

## Acknowledgements

We are grateful to the people of the study areas and the field staff who successfully conducted the project. The data can be obtained from icddr,b through their data sharing policy (http://www.icddrb.org/component/content/article/10003-datapolicies/1893-data-policies).

## Funding

The study was supported by the core grants to the icddr,b, by the Governments of Bangladesh, Canada, Sweden, and the UK, and also by the Bill & Melinda Gates Foundation in grants to the DOVE project of Johns Hopkins Bloomberg School of Public Health (OPP1148763).

## Competing interests

None declared.

## Ethical approval

The project, including verbal informed consent, was approved by the Ethical Review Committees of the icddr,b, Dhaka, Bangladesh and the WHO. Verbal informed consents were obtained from all participants, which were documented in the vaccine record book.
